# Effect of exercise on quality of life among menopausal women

**DOI:** 10.6026/973206300210155

**Published:** 2025-02-28

**Authors:** Bhuvaneshwari D., Sathiyalatha Sarathi

**Affiliations:** 1 Department of Nursing, Bharath Institute of Higher Education and Research, Chennai, Tamilnadu, India; 2 Department of OBG Nursing, Sree Balaji College of Nursing, Chennai, Tamilnadu, India

**Keywords:** Weight bearing exercise, breathing exercise, quality of life, menopause

## Abstract

Menopause is one of the most important phases of a woman's life. Therefore, it is of interest to conduct a randomized-controlled trial approach and three hundred post-menopausal volunteers were split into two groups randomly and quality of life was evaluated using the Nottingham Health Profile (NHP). The exercise group engaged in a program consisting of submaximal weight-bearing and breathing exercises for eight weeks, five times a week. The quality of life in the two groups was compared after eight weeks. The NHP's quality of life improved in the exercise group, with a statistically significant difference (P < 0.05). Thus, an 8-week regimen of consistent, supervised exercise could enhance the quality of life for post-menopausal women.

## Background:

Women are one of the most vital pillars of the family and society and addressing their needs is crucial to the community's overall well-being. Menopause is one of the most important phases of a woman's life [1]. Everywoman experiences the menopause, which is the time in her life when her menstrual cycle permanently ends. This stage, which typically lasts from 40 to 60 years of age, is linked to changes in hormones, the body and the mind [2]. Early symptoms include hot flashes, impatience, sleep disturbances, exhaustion, anxiety and loss of focus, depending on the ovarian function issue and consequently, the lack of estrogen in the postmenopausal era. The decrease of estrogen's protective properties in later life increases the risk of coronary artery disease and incidence rate of osteoporosis. These postmenopausal symptoms have a negative impact on women's quality of life. Women experience the pathological changes they encounter during the postmenopausal phase for one-third of their life, if we believe that menopause age starts at age 50 [3]. The World Health Organization had previously predicted that 1.2 billion women would be over 50 by 2003. Consequently, even though the menopause appears to be a normal process, it is a time that needs to be monitored and managed. Improving life quality is one of the goals of universal health in the twenty-first century [4].

The problems associated with menopause, a physiological occurrence, affect women's quality of life. To lessen menopausal symptoms and enhance quality of life at this time, a number of strategies, including hormone therapy and non-pharmacological therapies, are advised. One common treatment for menopause is hormone replacement therapy or HRT. It is now commonly acknowledged that exercise can help prevent postmenopausal symptoms as an additional therapeutic strategy. It is possible to dramatically reduce postmenopausal symptoms by encouraging middle-aged women to develop the habit of regular exercise. Previous studies have demonstrated that postmenopausal women who reside in urban areas tend to be more sedentary. Weight gain is a regular occurrence among people living in rural areas because of uneven and single-type diet. Regular exercise and burning extra calories reduce weight gain, which is influenced by age, inactivity and hormone medications taken during menopause [5]. Exercise affects bone density, muscle strength and postmenopausal symptoms in women going through this stage [6]. Therefore, it is of interest to conduct a randomized-controlled trial approach and three hundred post-menopausal volunteers were split into two groups randomly and quality of life was evaluated using the Nottingham Health Profile (NHP).

## Methodology:

In a few chosen locations in Gudur, Andra Pradesh, India, a mixed-method study was carried out. For the study, menopausal women were chosen using a straight forward random sample procedure. In the initial round, ten communities were chosen by lottery. The study included roughly 15-20 menopausal women from each town. Power analysis and the findings of a pilot research were used to determine the sample size. Before the study started, power analysis was also used to assess the sample size. Each group's anticipated sample size was 150.Three hundred menopausal women made up the study's overall sample. 150 of these were assigned to a control group and another 150 to a study group. The sample was chosen based on the following inclusion criteria: women who practiced yoga at least five days a week; age 45-55 years; persistent menopausal symptoms; and a permanent, natural cessation of menstruation. Gynecological issues such uterine fibroids, dysfunctional uterine hemorrhage, or prolapsed uterus, hormone replacement therapy and current medical treatment for menopausal symptoms were among the exclusion criteria.

Following sample selection, baseline demographic and quality of life data were gathered from every study participant. Following the pretest, the study group's menopausal women received education on menopause, breathing techniques and weight-bearing exercises. At weeks 6, 12 and 18, the researcher used the same questionnaire to measure quality of life and after each test, reinforcement sessions were conducted. Two sections made up the questionnaire used in this investigation. Age, religion, marital status, family type, availability of a support system, menarche age, parity and length of time to menopause were among the demographic variables covered in Section 1. The Nottingham Health Profile (NPH) was included in Section 2. There were 38 yes/no questions about health systems in the NPH. Each of the six NPH sub-sections-physical mobility, pain, sleep, energy, social isolation and emotional reactions-had a different number of questions and a varied score. The cumulative score for each sub-section should be at least 0 (best health) and at most 100 (worst health). A summary score was absent.

## Results:

SPSS version 14.0 was used to conduct the statistical analysis. The means ± standard deviation (SD) was used to report all data. The differences between the exercise and control groups were compared using an independent sample t-test, whereas the differences between the same group before and after exercise were compared using a paired sample t-test. For more than two groups, the ANOVA test was employed and for continuous and categorical data, the Chi-Square test was utilized, accordingly. P<0.05 was regarded as statistically significant for all analyses. According to NHP, there was no statistically significant difference, as Table 1 illustrates, suggesting that quality of life had improved prior to exercise. Six sub-items of the NHP indicate that the exercise group's quality of life has improved, as seen by the survey results Table 2, which demonstrate substantial decreases in the scales of all the items. After eight weeks, there were no discernible differences in the control group's quality of life. According to NHP, there was a statistically significant difference in the exercise group at the conclusion of the eight weeks, suggesting that their quality of life had improved.

## Discussion:

Women go through the postmenopausal phase for nearly one-third of their lives after menopause, which is a normal occurrence that typically occurs around the age of 50. The physical, psychological and social issues that begin with menopause cause the quality of life to deteriorate during the postmenopausal phase. Numerous studies stress the importance of combining HRT with an exercise regimen to get the most out of the treatment [7]. Numerous studies show that postmenopausal symptoms have a detrimental impact on quality of life. According to Luis Cobero, president of the Spanish Gynecology and Obstetrics Association, postmenopausal women's quality of life is enhanced not only by hormone replacement therapy (HRT) but also by dietary changes, lifestyle adjustments and consistent exercise. In order to determine the consequences of osteoporosis in 50 postmenopausal women, Guzeloglu used NHP. She found that the longer the postmenopausal period, the greater the degradation in quality of life. Guzeloglu underlined that the NHP scale had a fairly high degree of reliability and could be used to assess postmenopausal women's quality of life. One intervention trial found that a regular, controlled six-week exercise program could improve postmenopausal women's quality of life and fitness level.

Elavsky discovered a relationship between physical exercise and menopausal symptoms, as well as between physical self-worth and positive effect. Higher levels of these factors were linked to higher menopause-related quality of life scores [8]. Barbara *et al.* conducted a study and her results clearly show that 12-week moderate-intensity aerobic exercise does not reduce VMS, but it may slightly improve midlife, sedentary women's sleep, insomnia and depression [9]. The results of a meta-analysis by Hong *et al.* who conducted a study in China, show that mind-body training benefits peri-menopausal and postmenopausal women's bone mineral density, sleep quality, anxiety, depression and fatigue [10]. Strength training can help increase bone density, strength, physical activity and hormone and metabolic levels, according to a study conducted in Spain by Maria *et al.* Since several forms of workouts yield the same advantages, the evidence about the best kind of strength training is still unclear [11]. Jolanta *et al.* conducted a study in Poland and found a substantial correlation between regular exercise for 12 weeks and improvements in mental and physical health. Women who are sedentary should think about changing their lifestyle to incorporate physical activity since it improves their quality of life [12]. According to a study conducted in Finland by Jaana, aerobic exercise for six months may help inactive women have fewer menopausal symptoms, including mood swings, irritability and night sweats. We assessed the quality of life using NHP as well. All of the NHP sub-items showed an improvement in life quality in the exercise group. Nguyen *et al.* suggested that well-designed studies are needed to confirm the effect of exercise on quality of life in women with menopausal symptoms [13].

## Conclusion:

Regular and controlled exercise for eight weeks could improve post-menopausal women's quality of life. The long-term impact of exercise on quality of life can be assessed if these people can develop the habit of exercising as a lifestyle. Such an assessment will require studies that use data from the same cohort in later years.

## Figures and Tables

**Table 1:** Mean quality of life scores in the exercise and control groups before exercise

**Subarea**	**Exercise group Mean & SD**	**Control group**	**P-value by t-test**
Energy level	7.77 (4.1)	8.01 (2.95)	0.7
Pain	22.39(6.21)	23.2(7.32)	0.7
Emotional reaction	25.22(7.45)	26.13(6.35)	0.7
Sleep	21.65(6.25)	14.04(5.5)	0.08
Social isolation	13.55(6.02)	14.02(5.23)	0.7
Physical abilities	22.3(5.11)	23.2(7.41)	0.6
Total	112.88(35.14)	108.6(34.76)	0.7

**Table 2:** Mean quality of life scores in the exercise and control groups after exercise

**Subarea**	**Exercise group Mean & SD**	**Control group**	**P-value by t-test**
Energy level	11.60(3.96)	8.11 (3.75)	0.001
Pain	30.29(6.21)	21.2(6.32)	0.001
Emotional reaction	33.18(8.35)	25.13(7.35)	0.001
Sleep	18.65(8.25)	13.04(6.5)	0.001
Social isolation	12.55(7.02)	13.02(6.23)	0.001
Physical abilities	21.2(9.12)	22.2(6.41)	0.001
Total	127.47(42.91)	102.7 (36.56)	0.001
